# High Prevalence of Depression and Anxiety in Patients with Chronic Respiratory Diseases Admitted to Intensive Care in a Low-Resource Setting

**DOI:** 10.3390/arm93030012

**Published:** 2025-06-02

**Authors:** Amun Mustafa, Asifa Karamat, Wajeeha Mustansar Toor, Tehmina Mustafa

**Affiliations:** 1 Department of Medicine, Fatima Memorial Hospital College of Medicine and Dentistry, Lahore 54000, Pakistan; amunmustafa2@gmail.com; 2 Department of Pulmonology, Gulab Devi Teaching Hospital, Lahore 54000, Pakistan; asifa.karamat@gmail.com (A.K.); drwajeeha_toor@hotmail.com (W.M.T.); 3 Center for International Health, Department of Global Public Health and Primary Care, University of Bergen, 5020 Bergen, Norway; 4 Department of Thoracic Medicine, Haukeland University Hospital, 5021 Bergen, Norway

**Keywords:** depression, anxiety, chronic respiratory diseases, intensive care, Hamilton Depression Rating Scale

## Abstract

**Highlights:**

**Main findings**:

- Depression and anxiety were highly prevalent among ICU patients with chronic respiratory diseases (CRDs), with over 80% experiencing depressive symptoms and up to 100% experiencing anxiety.
- Severe depression was notably more frequent in female patients and those of older age, while anxiety symptoms showed a strong correlation with depression and were more frequent in females.

**Implications of the main findings:**

- Routine mental health screening in critical-care settings for patients with CRDs is essential for early detection and holistic management.
- Integrating psychiatric support into ICU care pathways may improve patient outcomes and reduce the long-term burden of untreated psychological comorbidities.

**Abstract:**

Background: Depression and anxiety are common in patients with chronic respiratory diseases (CRDs), but their prevalence in intensive care settings, particularly in low-resource regions, remains underexplored. Objective: To assess the prevalence and severity of depression and anxiety in patients with CRDs admitted to an intensive care unit (ICU) and identify associated factors. Methods: A cross-sectional study was conducted at Gulab Devi Teaching Hospital, Lahore, Pakistan. Adult patients with CRDs admitted to the ICU were assessed using the Hamilton Depression Rating Scale. Statistical analyses included Fisher’s exact test, Mann–Whitney/Kruskal–Wallis tests, and logistic regression. Results: Depression was highly prevalent across all CRD categories: 83%, 89%, 84%, and 93% in obstructive, restrictive, infectious, and other respiratory disease categories, and severe depression in 16%, 18%, 14%, and 37%, respectively. Anxiety symptoms were also widespread (77–100%), with no significant differences across disease groups. Depression was significantly associated with older age (*p* < 0.001, OR 1.08) and anxiety symptoms (*p* < 0.001, OR 47.07). Female gender was linked to anxiety (*p* = 0.034, OR 4.17). Conclusion: The high burden of depression and anxiety in ICU patients with CRDs underscores the need for routine psychiatric screening and integrated mental health care in critical-care settings.

## 1. Introduction

Chronic respiratory diseases (CRDs) impose a significant global health burden, contributing substantially to morbidity and mortality [1,2]. Beyond their physiological impact, CRDs also exact a heavy psychological toll, consistent with the biopsychosocial model of chronic illness [3]. For instance, in COPD outpatients, reduced forced expiratory volume in one second (FEV_1_) and long-term oxygen dependence are strongly associated with depressive symptoms [4], whereas effective coping strategies and robust social support can buffer against emotional distress [5]. Depression and anxiety are highly prevalent in advanced CRD, affecting an estimated 40–80% of patients with depression and 50–75% with anxiety [6,7], and are linked to longer hospital stays, higher readmission rates, increased treatment costs [8,9], and diminished quality of life [10,11]. Despite this burden, head-to-head comparisons of mood disorder prevalence across COPD and other CRD subtypes are rare, and virtually no data exist from low-resource ICU settings such as Pakistan. Moreover, in critically ill patients, overlapping somatic and psychiatric presentations [12] and ICU-specific stressors, sleep disruption, sensory deprivation, and social isolation [13] mask or amplify these symptoms, leaving the true scope of depression and anxiety in this population largely unknown. This gap underscores the need for further studies which systematically assess the prevalence and severity of mood disturbances across CRD subtypes in a resource-constrained ICU environment.

We therefore conducted a cross-sectional study in the ICU of a tertiary hospital in Lahore, Pakistan, to (i) quantify the prevalence and severity of depression and anxiety across CRD subtypes and (ii) identify demographic and clinical factors associated with these mood disturbances.

We hypothesized that patients with CRDs admitted to the ICU would have a high prevalence of depression and anxiety and the prevalence would vary across CRD subtypes.

## 2. Materials and Methods

### 2.1. The Study Design and Participants

We conducted a cross-sectional, single-center observational study in the medical ICU at the Gulab Devi Teaching Hospital (GDH), Lahore, Pakistan, between January and December 2022. GDH is a private-sector, not-for-profit tertiary care hospital, catering to the needs of the poor population. Participants were eligible if they had a confirmed diagnosis of CRD for at least 12 months, were admitted to the ICU, and were able to provide informed consent. We excluded any patient with a documented pre-existing major psychiatric disorder (such as bipolar disorder or schizophrenia), those already on psychotropic drugs, those with cognitive impairment severe enough to preclude reliable response to the questionnaire, and individuals experiencing active substance intoxication or withdrawal. The family history of psychiatric disorders was not asked for.

### 2.2. Procedures

Screening was conducted by trained physicians using the Hamilton Depression Rating Scale (HDRS) to determine the prevalence and quantify the severity of depressive symptoms in patients. The HDRS includes 17 questions and can have a maximum score of 52 [14]. The following score categorization was used: normal/no depression 0–7; mild depression 8–16; moderate depression 17–23; severe depression > 24. Questions 10 and 11 were used to study psychic and somatic anxiety symptoms.

Patients were categorized into obstructive, restrictive, infectious, or other based on the clinicians’ assessment.

### 2.3. Data Management and Statistical Analysis

All data was recorded on the predesigned questionnaires without identifiable patient information. Data was entered into IBM Statistical Package for the Social Sciences (SPSS) Version 29 and Microsoft Excel 365 for data cleaning and analysis. Descriptive statistics were presented, and group differences were compared using Fisher’s exact test for categorical variables and non-parametric Mann–Whitney or the Kruskal–Wallis tests for continuous variables [15]. The general significance level was set to 0.05. Due to the larger number of tests, we used the Bonferroni adjustment, leading to the marginal levels of 0.008 for 6 comparisons between the respiratory disease categories. Multiple logistic regression was used to analyze factors associated with depression and symptoms of anxiety.

### 2.4. Ethical Approval

The study was carried out following the rules of the Declaration of Helsinki of 1975, which was revised in 2013. The study was approved by the Institutional Review Board, Al. Aleem Medical College & Gulab Devi Educational Complex Lahore (GDEC/235/19) and the Regional Committee for Medical and Health Research Ethics, Western-Norway (2020/152522/REK vest). All the participants provided informed written consent.

## 3. Results

### 3.1. Demographic Characteristics and HDRS Scores by Respiratory Disease Categories

In total, 185 cases were included in the analysis. Table 1 shows the demographic characteristics of patients. The median age of patients in the obstructive and restrictive disease groups was higher (55 and 56 years, respectively) as compared to the patients in the infection and other group (43 and 45 years, respectively) (*p* = 0.001). In the obstructive disease group, there were more males, while there were more females in all other groups (*p* = 0.001). The patients in the obstructive disease group had the longest duration of illness (median 3 years), followed by the restrictive disease group (median 2 years), while the infection and the other group had a shorter illness duration (median 1.3 and 1.4 years, respectively, *p* = 0.001). The duration of admission was the longest among the infection group. The median total HDRS score among the various groups was not significantly different (*p* = 0.021).

### 3.2. Prevalence of Depression and Anxiety by Respiratory Disease Categories

**Figure 1:**
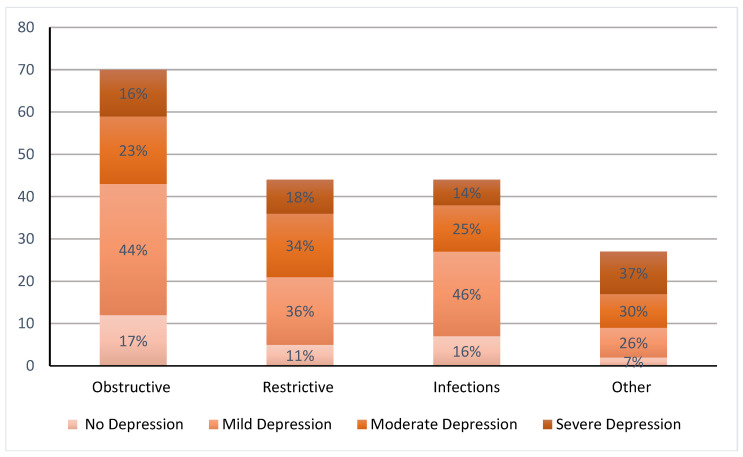
Prevalence and the severity of depression among the various categories of chronic respiratory dis-eases. There was no difference between the prevalence of depression and the severity of depression between the different disease categories.

The prevalence of depression was high: 83%, 89%, 84%, and 93% among the obstructive, restrictive, infection, and other groups, respectively (Figure 1). The majority had mild depression with a prevalence of 44%, 36%, 46%, and 30%, while the prevalence of severe depression was 16%, 18%, 14% and 37% among the obstructive, restrictive, infection, and other groups, respectively. There was no difference between the prevalence of overall depression (*p* = 0.58) or the severity of depression (*p* = 0.28) between the different disease categories.

**Figure 2:**
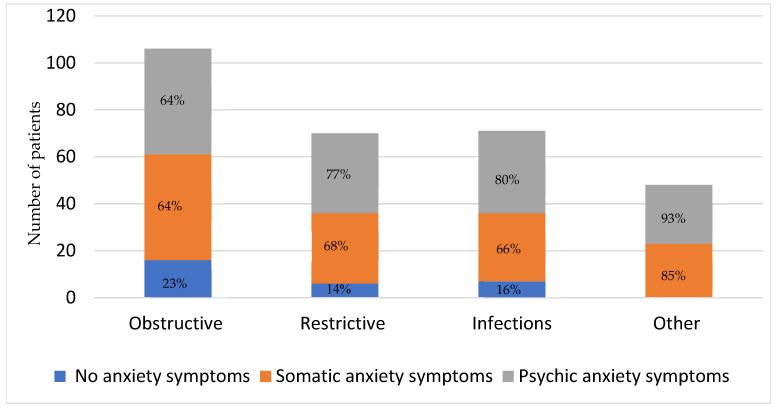
Prevalence of somatic and psychic anxiety symptoms among the various categories of chronic respiratory diseases. There was no statistically significant difference in the prevalence of anxiety symptoms between the different disease groups.

The prevalence of anxiety symptoms was high: 77%, 86%, 84%, and 100% among the obstructive, restrictive, infection, and other groups, respectively (Figure 2). There was no statistically significant difference in the prevalence of somatic (*p* = 0.24) or psychic anxiety (*p* = 0.02) symptoms between the different disease groups. There was also no difference in the prevalence of somatic or psychic anxiety symptoms as compared to no anxiety between the different groups (*p* = 0.04).

There was a significant correlation between the presence of any anxiety and depression symptoms (Pearson correlation coefficient 0.510, *p* < 0.001).

### 3.3. Prevalence of Depression and Anxiety by Gender and Age

**Figure 3:**
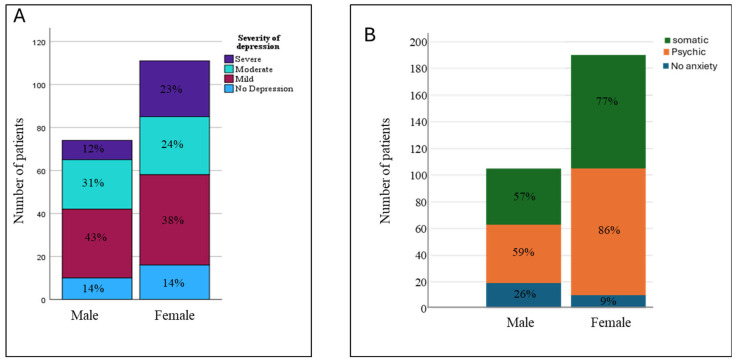
Comparison of the prevalence of depression (**A**) and anxiety (**B**) between male and female patients. Severe depression was more frequent among females as compared to males (*p* = 0.05). The prevalence of any anxiety, psychic anxiety, and somatic anxiety symptoms was higher among females as compared to males (*p* < 0.004).

There was no difference in the prevalence of any depression between males (86%) and females (85%) (Figure 3A). However, severe depression was more frequent among females (23%) as compared to males (12%) (*p* = 0.05). The prevalence of any anxiety, psychic anxiety, and somatic anxiety symptoms was higher among females (91%, 85%, 76%) as compared to males (74%, 59%, 57%) (*p* < 0.004) (Figure 3B).

**Figure 4:**
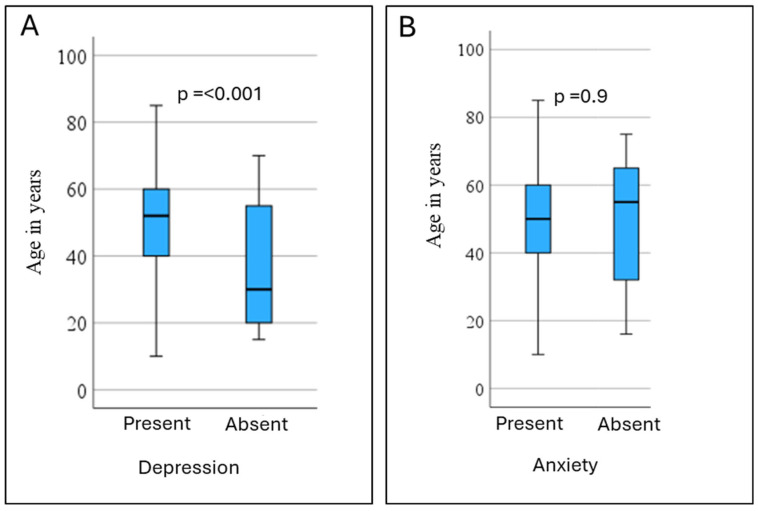
Comparison of the prevalence of depression (**A**) and anxiety (**B**) according to age. Patients with depression were older (*p* < 0.001), while there was no difference in the ages of patients with or without anxiety.

Patients with depression were older as compared to the patients with no depression (median age 52 and 30 years, respectively) (*p* < 0.001) (Figure 4A). There was no difference between the ages of patients with mild, moderate, or severe depression. Similarly, there was no difference in the ages of patients with or without anxiety (median age 50 and 55 years, respectively) (Figure 4B) or with psychic or somatic anxiety.

### 3.4. Factors Associated with Depression or Anxiety

Table 2 shows the comparison of the patients with and without depression and anxiety. Patients with depression had higher age, and more females had anxiety. The duration of illness, the duration of admission in the hospital, and the respiratory disease category were not different between patients with or without depression or anxiety symptoms.

By using multiple logistic regression, higher age (OR 1.08, CI 1.04–1.14) and presence of anxiety symptoms (OR 47.077 (CI 9.86–224.69) were found to be associated with depression. Being a female (4.17 (1.11–15.65) and the presence of depression (46.89 (10.41–211.25) were associated with anxiety symptoms (Table 3)

## 4. Discussion

In our ICU cohort of CRD patients from a low-resource setting, we found an alarmingly high prevalence of depression (83–93%) and anxiety (77–100%) with no significant variation across obstructive, restrictive, infectious, or other CRD subtypes. While previous outpatient studies reported mood disorder rates of roughly 37–71% for depression and 51–75% for anxiety in advanced COPD [7] and a 46% depression prevalence among general CRD inpatients in a Moroccan pneumology ward [16], our substantially higher figures likely reflect the added burden of critical-illness stressors—sensory deprivation, sleep disruption, diagnostic uncertainty, and social isolation that are ubiquitous in the ICU [13]. Moreover, more severe disease in ICU settings appears to amplify psychological distress: Lacasse et al. noted 57% depression in oxygen-dependent COPD [4], and Kunik et al. observed anxiety and depression rates of 80% in patients with chronic breathing disorders [6]. Mechanistically, hypoxemia and systemic inflammation can dysregulate neuroendocrine stress pathways, while dyspnea itself may condition fear responses via limbic activation [17]. The co-occurrence of depression and anxiety establishes a vicious cycle, worsening disease severity, prolonging hospital stays, driving up treatment costs, and increasing mortality [8,9], thereby underscoring the imperative for early detection and integrated psychiatric management within critical-care pathways to enhance long-term patient outcomes and cost-effectiveness.

Although most patients in our cohort experienced mild depressive symptoms, the prevalence of severe depression varied substantially, from 16% in the obstructive group to 37% among the “Other” CRD patients. This pattern may reflect heterogeneous pathophysiology and prognostic uncertainty in conditions such as bronchiectasis or overlap syndromes. In contrast, our infectious CRD subgroup, marked by acute inflammation and fever, exhibited lower rates of severe depression but similarly elevated anxiety, suggesting that instantaneous mortality threat, rather than long-term disease adaptation, drives their distress [7,18]. The lack of statistically significant differences in somatic versus psychic anxiety across groups further underscores that both neurovegetative and cognitive dimensions of anxiety are pervasive in critically ill CRD patients.

A previous study from Karachi, Pakistan, studied the prevalence of depression and anxiety in patients with COPD and found a lower prevalence of depression and anxiety, 51% and 20%, respectively, as compared to our study [19]. Our work differs from this study in three key respects. Husain et al. conducted assessments in an outpatient clinic, whereas our cohort comprises patients admitted to the ICU, representing a more severe clinical spectrum. Second, we employed the structured 17-item HDRS and its anxiety subscale, and thirdly, we studied the different categories of CRD, while Husain et al. studied only COPD. These methodological enhancements and broader disease scope underscore the critical importance of comprehensive, setting-specific mental health evaluation in CRD populations.

We observed that women reported significantly higher anxiety and severe depression, corroborating earlier studies suggesting hormonal and societal influences. For instance, in a review, female COPD patients are reported to be more likely to experience anxiety and depression symptoms [11]. Similarly, an earlier COPD study has reported a higher risk of depression among women [20]. Estrogen and progesterone fluctuations modulate the hypothalamic–pituitary–adrenal axis, heightening stress reactivity [21,22]. Psychosocially, female patients often carry greater caregiver responsibilities, intensifying anticipatory worry about dependents when critically ill [23]. Finally, gender differences in symptom reporting may contribute to the higher observed anxiety. Male patients, nonetheless, exhibited a considerable burden of both anxiety and depression. This underscores that psychological comorbidity is a critical concern for all CRD patients, regardless of sex. Clinicians should therefore proactively screen for and address these disorders in both men and women to optimize patient outcomes.

Older age was significantly associated with depression; patients with depression had a median age of 55 years versus 35 years in those without, whereas anxiety showed no age relationship. This aligns with population data indicating that depression risk increases with age due to factors such as social isolation, role loss, and bereavement [12,24]. In CRD specifically, older patients often endure longer disease duration, more pronounced functional impairment, and greater symptom severity, all of which have been linked to higher depression rates [10]. Together, these findings suggest that the combined impact of advancing age, cumulative disease burden, and age-related social challenges contributes to the elevated prevalence of depression among older ICU patients with CRD.

We found a significant correlation between anxiety and depression and mutual risk factors for each other. This aligns with the literature on CRD comorbidity, where co-occurring mood disorders synergistically worsen clinical outcomes [5,11,25,26]. Biologically, inflammation and hypoxemia common to all CRDs can dysregulate neuroendocrine pathways, while psychosocial factors, such as loss of autonomy and concerns about prognosis, compound emotional distress [5,11,17,25,26]. Clinically, this comorbidity predicts longer ICU stays, higher readmission rates, and increased mortality, advocating for integrated psychiatric evaluation alongside respiratory care, particularly in older populations.

Despite a 14–37% prevalence of severe depression in our ICU cohort, none of these patients were receiving mental health treatment. This treatment gap likely reflects limited recognition of psychiatric comorbidity by both health care providers and patients [25,27], and a pervasive stigma that deters individuals from acknowledging emotional distress and seeking care [28]. Consequently, anxiety and depressive symptoms frequently present as somatic complaints, such as dyspnea or fatigue, further delaying appropriate diagnosis and intervention [29]. To overcome these barriers, mental health screening and treatment must be integrated into routine hospital workflows, ensuring that psychological as well as physical needs are addressed in tandem.

As the ICU environment itself compounds psychological vulnerability [13,30], some improvement can, therefore, be expected in some patients following discharge without psychological intervention. However, persistent depression and anxiety often stem from the chronic burden of respiratory impairment, loss of autonomy, and uncertainty about long-term prognosis [5]. Left unaddressed, these psychiatric comorbidities impede rehabilitation, diminish quality of life, and increase hospital readmission rates [8]. Accordingly, ICU teams should be trained to recognize early signs of distress, deliver brief psychosocial interventions (e.g., coping-skills coaching, family liaison), and coordinate timely referrals to mental health specialists. Embedding psychiatric care within critical-care pathways will ensure that both the emotional and physiological dimensions of recovery receive the attention they require.

Our study’s primary strength lies in its real-world focus on a low-resource ICU, capturing mood disturbances across a diverse spectrum of CRD subtypes under routine clinical care. However, its cross-sectional, single-center design limits causal inference and generalizability. Key variables such as body mass index and socioeconomic status, known to influence psychosocial well-being, were inconsistently recorded and thus excluded, which may have introduced residual confounding. Finally, while the HDRS reliably quantifies symptom severity, it does not provide a view of functional impairment and quality-of-life outcomes. Future longitudinal, multicenter studies should integrate comprehensive psychosocial assessments and explore the impact of in-ICU psychological interventions on long-term recovery.

## 5. Conclusions

Our study reveals an alarmingly high prevalence of both depression (83–93%) and anxiety (77–100%) among ICU patients with CRD, with no meaningful differences across obstructive, restrictive, infectious, or other CRD subtypes. These findings underscore that, beyond the pathophysiology of individual disease categories, the critical-care environment itself exerts a profound impact on patients’ psychological well-being. Implementing routine screening for depression and anxiety on ICU admission and offering brief psychosocial support, with timely mental health referrals, could improve patient recovery and reduce costs. Future multicenter, longitudinal research should assess the long-term impact of these interventions and identify key biological and social risk factors.

## Figures and Tables

**Table 1:** Demographic characteristics of patients and Hamilton Depression Rating Scale scores.

	Respiratory Disease Categorization				
	Obstructive	Restrictive	Infections	Other	*p*-Value
	(*n* = 70)	(*n* = 44)	(*n* = 44)	(*n* = 27)	
Age—years median (IQR)	55 (14)	56 (22)	43 (28)	45 (24)	0.001
Sex					0.001
- Male	50	13	9	2	
- Female	20	31	35	25	
Illness duration—years median (IQR)	3 (4)	2 (4)	1.3 (1)	1.4 (1)	0.001
Admission duration—days median (IQR)	7(7)	7 (7.5)	17 (8)	7 (4)	0.031
HDRS total score median (IQR)	14 (9)	18 (12)	14 (13)	20 (15)	0.021

**Table 2:** Comparison of patients with and without depression and anxiety.

	Depression			Anxiety		
	Yes	No	*p* Value	Yes	No	*p* Value
Age—years median (IQR)	55 (20)	30 (35)	<0.001	50 (20)	55 (35)	0.1
Sex, *n*			0.45			0.04
- Male	64	10		55	19	
- Female	95	16		101	10	
Respiratory disease category, *n*			0.02			0.08
Obstructive (*n* = 70)	58	12		54	16	
Restrictive (*n* = 44)	39	5		38	6	
Infections (*n* = 44)	37	7		37	7	
Other (*n* = 27)	25	2		27	0	
Illness duration—years median (IQR)	2 (3)	1.4 (1)	0.2	1.9 (3)	2 (2.5)	0.4
Admission duration—days median (IQR)	7 (7)	5.5 (4.7)	0.6	5(7)	7 (8)	0.8

**Table 3:** Factors associated with depression and anxiety by multiple logistic regression analysis.

	Depression		Anxiety	
	Odds Ratio (95% CI)	*p* Values	Odds Ratio (95% CI)	*p* Values
Age	1.09 (1.04–1.14)	<0.001	0.98 (0.94–1.02)	0.360
Sex	0.75 (0.14–4.02)	0.733	4.17(1.11–15.65)	0.034
Disease category				
- Obstructive	(ref)	0.557	(ref)	0.964
- Restrictive	1.13 (0.21–6.20)	0.886	1.19 (0.31–4.55)	0.793
- Infections	3.33 (0.54–20.61)	0.195	0.78 (0.20–3.17)	0.735
- Other	2.45 (0.28–21.77)	0.422	19 (0-)	0.998
Illness duration—years	1.08 (0.90–1.31)	0.406	1.01 (0.87–1.17)	0.886
Admission duration—days	1.04 (0.94–1.16)	0.437	1.00 (0.97–1.04)	0.830
Psychic or somatic anxiety	47.07 (9.86–224.69)	<0.001		
Depression			46.89 (10.41–211.25)	0.000

## Data Availability

The data presented in this study are available on request from the corresponding author.
